# Porous Au/Ti Bilayer Thin-Film Getters Based on Black Silicon for MEMS Vacuum Packaging

**DOI:** 10.3390/mi17050520

**Published:** 2026-04-24

**Authors:** Kunwei Zhao, Tianyou Chen, Yuelong Liu, Ji Fan

**Affiliations:** National Gravimetry Laboratory, School of Physics, Huazhong University of Science and Technology, Wuhan 430074, China; zhaokunwei@hust.edu.cn (K.Z.); chentianyou@hust.edu.cn (T.C.); fanji@hust.edu.cn (J.F.)

**Keywords:** MEMS vacuum packaging, thin-film getter, black silicon, porous structure

## Abstract

Porous thin-film getters are extensively utilized in the field of MEMS vacuum packaging. Nevertheless, their effectiveness is frequently constrained by the comparatively modest effective surface area of conventional planar structures. In this work, a porous Au/Ti thin-film getter based on a three-dimensional black silicon scaffold is developed to enhance the effective surface area and improve gettering performance. The fabrication of black silicon nanostructures is achieved through an SF_6_/O_2_-based inductively coupled plasma (ICP) etching process, followed by the deposition of Au/Ti bilayer films by DC magnetron sputtering. The morphological evolution of the Ti film on the nanostructured substrate and the activation behavior of the Au/Ti bilayer are systematically investigated using scanning electron microscopy (SEM) and X-ray photoelectron spectroscopy (XPS). The results demonstrate that the shadowing effect during sputtering leads to the formation of a porous film with increased surface roughness and an open structure. XPS analysis demonstrates that there is a significant increase in the oxygen content on the surface at higher activation temperatures. This suggests that effective sorption capability is achieved following activation. In comparison with planar substrates, the three-dimensional black silicon scaffold has been demonstrated to promote the formation of a more open and functional structure. The results obtained from this study indicate that the proposed fabrication strategy offers a feasible and MEMS-compatible approach for the construction of porous thin-film getters, thereby enhancing their effective surface area.

## 1. Introduction

Micro-electro-mechanical systems (MEMS) devices are extensively utilized in a multitude of applications due to their inherent miniaturization potential, seamless integration compatibility, exceptional electrical performance, and optimal suitability for large-scale manufacturing. However, devices such as accelerometers [1,2,3], gyroscopes [4], micro-radiation calorimeters [5,6], and radio-frequency (RF) MEMS [7,8] typically require a stable vacuum environment to enhance their performance. Vacuum packaging is employed to protect these devices from extreme conditions, thereby ensuring optimal functionality of their mobile components. Furthermore, damping effects have been shown to have a substantial impact on the quality factor (*Q*) of these devices, with the potential to result in performance degradation. To maintain low residual gas pressure throughout the lifetime of the device, getter materials are typically integrated into the cavity to absorb the gases generated by leakage, permeation and surface outgassing [9,10,11,12]. However, conventional sheet or bulk getters are incompatible with microscale packaging. In recent years, there has been significant attention given to thin-film getters that are integrated into micro-system cavities [13,14,15]. Thin-film getters are classified into two distinct categories, namely evaporated and non-evaporated, based on their respective operational mechanisms. Evaporated getters require a heating process to evaporate active metals for residual gas absorption, which may result in metal powder contamination. In contrast, non-evaporated getters maintain their morphology during activation, rendering them more compatible with devices that incorporate movable components.

Reactive metals, including titanium (Ti), zirconium (Zr), hafnium (Hf), vanadium (V), and yttrium (Y), are commonly employed as getter materials. These metals have a propensity to react with air, forming a passive surface layer. Prior to packaging, activation in a vacuum environment is therefore required [16,17]. Activation is defined as the process of subjecting metals to an annealing process, whereby the metals are heated to a specific temperature and held for a predetermined duration. In the context of the rapid advancement in the field of microsystems, the development of low-temperature activation techniques has emerged as a critical imperative [18]. Alloy and multilayer film getters have been shown to offer superior sorption performance in comparison with pure metals, and require lower activation temperatures. Wang et al. [19] demonstrated that the activation process of TiZrV can be initiated at temperatures as low as 150 °C, and can be enhanced by increasing the temperature. Bessouet et al. [20] reported that Y-Ti alloy films with a Y content exceeding 30% exhibit significant oxygen adsorption capability during annealing below 300 °C. In contrast, no oxygen adsorption was detected for pure Ti films under the same conditions. However, these alloy getter films often require customized targets or co-sputtering equipment. Additionally, precise control over the proportion of alloy ingredients is imperative for ensuring optimal sorption performance. Alternatively, multilayer thin-film getters are more convenient for industrial wafer-level packaging processes. It is important to note that Ti is compatible with complementary metal-oxide-semiconductor (CMOS) technology, which has led to an increase in its popularity in the field of MEMS vacuum packaging. Bourim et al. [21] showed that the addition of a ruthenium (Ru) seeding layer improves the sorption performance of multilayer getter films and lowers their activation temperature from 400 °C to 375 °C. Zhang et al. [22] deposited Pd/Ti bilayer films as a non-evaporable getter material using a sublimation method. The Pd layer prevents the Ti film from oxidation when exposed to air. This structure enables an activation temperature as low as 100 °C while maintaining high performance even after 15 activation cycles.

The miniaturization and integration requirements of microsystem vacuum encapsulation present a significant challenge, particularly with regard to internal cavities at the micrometer or millimeter scale. In such scenarios, the sorption capacity is constrained by the finite getter specific surface area. To address this limitation, porous films with high specific surface area and surface roughness have been proposed. Wu et al. [23] demonstrated that using an oblique evaporation angle can create a high-porosity film structure that enhances the sorption capacity of Ti thin-film getters. Cao et al. [24] proposed a Ti/Zr/V thin-film getter based on a three-dimensional nickel nanoarray, which achieved approximately three times the H_2_ sorption rate and twice the sorption capacity compared to silicon-based Ti-Zr-V films. Liu et al. [25] proposed a Si-Ni-AAO composite scaffold getter, which achieved a significantly larger specific surface area (250 times that of planar getters) and a greater sorption capacity (12 times that of planar getters). The utilization of substrates with three-dimensional structures has been identified as a viable strategy to address the constrained sorption capacity inherent in conventional two-dimensional getters. Despite the efficacy of contemporary methodologies, the intricate nature of their fabrication processes engenders elevated costs and challenges in the integration of getters into microsystem devices.

In this study, the development of a porous Au/Ti thin-film getter based on a three-dimensional black silicon scaffold is presented. This process enhances the effective surface area while avoiding the need for masking steps or complex alloy systems. Consequently, it demonstrates good compatibility with MEMS fabrication processes. The objective of this work is to systematically investigate the structural formation, morphological evolution, and activation behavior of the getter, and to evaluate its feasibility and compatibility for MEMS vacuum packaging applications. In this design, Au is employed as a protective layer due to exceptional chemical stability and its capacity to promote Ti activation through interdiffusion during annealing, thereby enabling effective gettering functionality.

## 2. Experiment

### 2.1. Preparation of Black Silicon Substrates

The experiments were conducted using 4-inch, 500-μm-thick, n-type, double-side-polished silicon wafers with a (100) crystal orientation. Prior to the fabrication of the black silicon structures, the wafers were meticulously cleaned in an ultrasonic bath using acetone and isopropyl alcohol to ensure the complete removal of any organic contaminants. The fabrication of the black silicon structures was achieved through utilization of an ICP etching system (PlasmaPro 100 Estrelas, Oxford Instruments, Abingdon, UK). This process does not necessitate the implementation of an additional masking layer, thereby facilitating the overall fabrication procedure. A modified Bosch etching process was employed, using SF_6_ and O_2_ as the reactive gases to form nanoscale needle-like structures on the silicon surface. Through the precise calibration of the ICP process parameters, a consistent black silicon morphology was successfully achieved. The detailed process parameters for black silicon formation are listed in Table 1. It is important to note that the black silicon structures investigated in this study were fabricated using a maskless ICP etching process without photoresist, resulting in the formation of nanostructures over the entire exposed silicon surface. In order to achieve region-selective patterning in a practical context, it is necessary to combine standard photolithography with ICP etching.

### 2.2. Deposition and Activation of Getter Films

The Au/Ti bilayer getter film was deposited using a DC magnetron sputtering system (FU-12PSB-CS, F. S. E Corporation, Taiwan, China). Prior to deposition, the chamber was evacuated to a base pressure of less than 3 × 10^−6^ Torr to minimize the influence of residual gases on film quality. The sputtering sources were high-purity titanium and gold targets (purity 99.999%). A 400 nm thick Ti film was first deposited on the black silicon substrate to serve as the active getter layer. Subsequently, a 20 nm thick Au film was deposited in situ as a protective layer without breaking the vacuum. The detailed deposition parameters of the getter film are summarized in Table 2.

Thermal activation of the getter was carried out in a vacuum wafer-bonding system (SB6 GEN2, SUSS MicroTec, Garching, Germany) to simulate the thermal conditions that are encountered during the vacuum packaging of microsystems. The activation temperature was varied from 200 °C to 450 °C to investigate the influence of temperature on the activation behavior of the Ti getter. During the activation process, the pressure within the chamber was maintained at a level of approximately 3.75 × 10^−2^ mTorr. The samples were maintained at the target temperature for a duration of one hour and subsequently permitted to cool naturally to ambient temperature under vacuum.

### 2.3. Characterization

The surface and cross-sectional morphologies of the black silicon substrate, as well as the Au/Ti getter film deposited on its surface, were characterized using SEM (ZEISS Sigma, Carl Zeiss AG, Oberkochen, Germany). Cross-sectional samples were prepared by mechanical fracture to examine the micro/nanostructures of black silicon and the conformal coverage of the deposited films. The elemental composition and chemical states of the film surfaces before and after activation were analyzed by XPS (AXIS-ULTRA DLD, Shimadzu Corporation, Kyoto, Japan).

## 3. Results and Discussion

**Figure 1:**
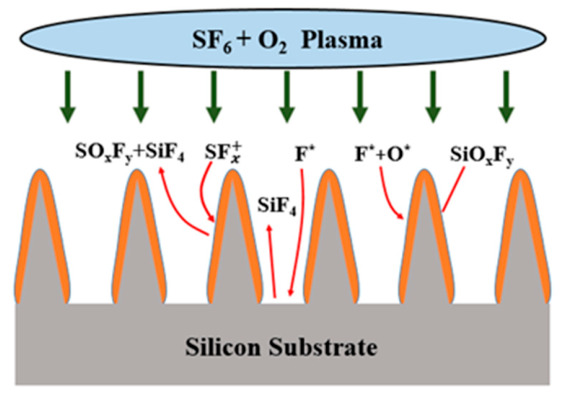
The formation mechanism of black silicon.

The formation of black silicon is primarily governed by the dynamic balance between the randomly formed silicon oxyfluoride (SiO_x_F_y_) masking layer on the silicon surface and the reactive ion etching induced by fluorine radicals (F^*^) in the plasma. The formation mechanism of black silicon is illustrated in Figure 1. Within the ICP environment, the plasma energy is significantly higher than the bond energies of S-F and O-O bonds, promoting the dissociation of SF_6_ and O_2_ to generate fluorine radicals (F^*^), oxygen radicals (O^*^), and ${SF}_{x}^{+}$ ions. The F^*^ radicals chemically react with silicon, resulting in the production of volatile SiF4 gas. Concurrently, F^*^ and O^*^ have the capacity to react with silicon, thereby forming a SiOxFy passivation layer on the surface, which functions to suppress further etching. In addition, the ${SF}_{x}^{+}$ ions can react with SiO_x_F_y_, generating SiO_x_F_y_ and SiF_4_, thereby partially removing the passivation layer. Due to the non-uniform distribution of the plasma, the SiO_x_F_y_ mask forms randomly across the sample surface. As a result, specific silicon locations are maintained during the primary stage of etching, subsequently functioning as “seeds” for the formation of nanoscale tips. It has been established that, during the etching process, the sidewalls of the structures are protected by the SiO_x_F_y_ passivation layer. This layer serves to reduce the impact of ion bombardment. In contrast, the bottom regions are continuously subjected to ion bombardment. This results in the repeated removal of the passivation layer. This, in turn, enables sustained vertical etching. This competitive mechanism between bottom etching and sidewall passivation results in a pronounced difference between the vertical and lateral etching rates. As the isotropic etching reaction continues, the diameter and depth of the pores between silicon sites gradually increase. When the pores expand to a sufficient size, lateral interconnection occurs between adjacent silicon sites. This process ultimately results in the formation of black silicon nanostructures with sharp geometric features on the surface. The black silicon fabrication method employed in this study, which is based on ICP, is consistent with previously reported SF_6_/O_2_ plasma processes. Steglich et al. [26] conducted a systematic investigation into the influence of process parameters on the morphology of black silicon nanostructures. In comparison with wet chemical etching methods such as MCE [27], the ICP-based method offers superior process controllability and better compatibility with MEMS fabrication processes. In this work, black silicon is not employed for optical optimization; rather, it functions as a three-dimensional scaffold for constructing a porous getter structure.

**Figure 2:**
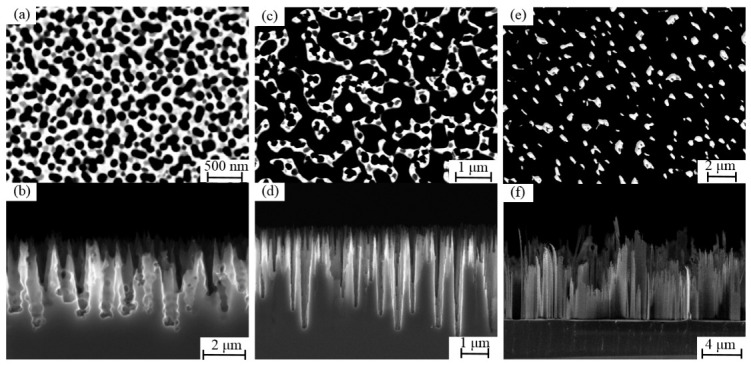
Surface (**a**,**c**,**e**) and cross-section (**b**,**d**,**f**) SEM of black silicon: (**a**) SF6 flow rate of 37 sccm; (**b**) SF6 flow rate of 42 sccm; (**c**) SF6 flow rate of 47 sccm.

The geometric morphology of the nanostructures is found to be strongly dependent on the selected etching parameters, including the SF_6_/O_2_ gas ratio, RF power, and chamber pressure. Among these, the SF_6_/O_2_ ratio plays a dominant role by regulating the balance between chemical etching and sidewall passivation. In this study, the RF power and chamber pressure were maintained at constant levels to ensure process stability, while the SF6 flow rate was systematically varied to optimise the nanostructure morphology. When the flow rate of the inhibitor gas O_2_ is maintained at a constant level, the SF6 flow rate exerts a significant influence on the evolution of the morphology during the process of black silicon formation. As shown in Figure 2, the surface and cross-sectional features of the black silicon were characterized by SEM. The O_2_ flow rate was maintained at a constant value of 60 sccm. When the SF_6_ flow rate was 37 sccm, the generation of fluorine radicals (F^*^) was inadequate, which restricted pore formation at the initial stage of etching. Consequently, the mean spacing between the protrusions was found to be approximately 100 nm (Figure 2a,b). An increase in the SF6 flow rate to 42 sccm resulted in a further enhancement of the etching process, leading to an increase in the average spacing between adjacent tips to approximately 500 nm (Figure 2c,d). The resulting nanoneedles exhibit a wider base and gradually taper towards the apex. This morphology has been shown to enhance the mechanical strength of the black silicon, while also providing a favourable substrate for the subsequent deposition of getter films. When the SF_6_ flow rate was further increased to 47 sccm, the reaction became more vigorous, resulting in the formation of sparse needle-like structures (Figure 2e,f). This morphology demonstrates inadequate mechanical strength and is consequently unsuitable for the subsequent deposition of getter films. Consequently, an SF_6_ flow rate of 42 sccm was selected for the subsequent processes. The geometry of black silicon nanoneedles has been observed to exhibit a dependence on the etching depth. During the ICP etching process, an increase in etching time results in a gradual vertical growth of the nanoneedles, with their height increasing in proportion to the etching depth. In addition to the lateral morphology control described above, a variation in etching depth further influences the overall structural evolution. At greater etching depths, the occurrence of partial coalescence between nanostructures or slight tip broadening may be observed. This is due to local etching effects and structural evolution. Such phenomena can result in variations in the overall porosity and structural connectivity. The relatively large etching depth (about 4 μm) is intentionally selected to increase the spacing between nanoneedles, thereby preventing the formation of a continuous metal film during subsequent deposition and enabling the formation of a three-dimensional porous structure with a higher effective surface area.

**Figure 3:**
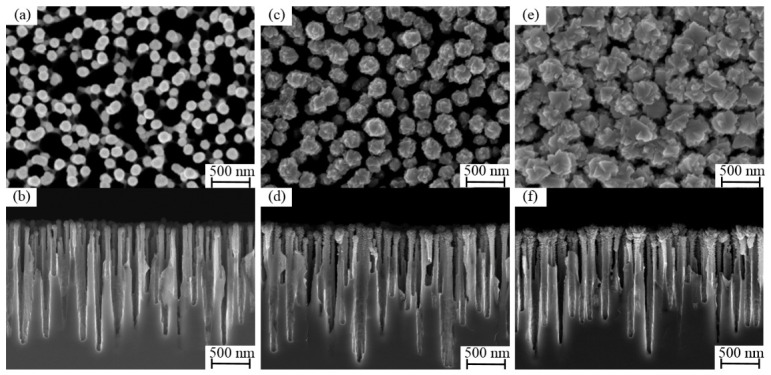
Surface (**a**,**c**,**e**) and cross-sectional (**b**,**d**,**f**) SEM images of getter films deposited on a black silicon substrate: (**a**) deposition time of 10 min; (**b**) deposition time of 20 min; (**c**) deposition time of 30 min.

**Figure 4:**
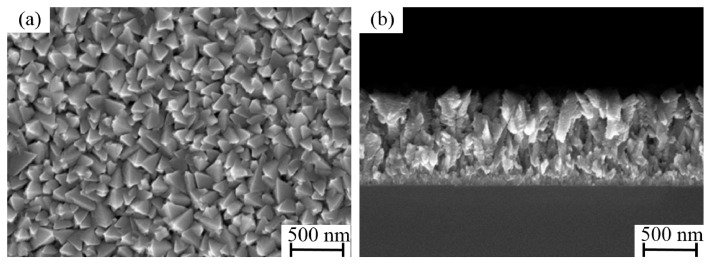
Surface (**a**) and cross-sectional (**b**) SEM images of getter films deposited on planar substrates.

**Figure 5:**
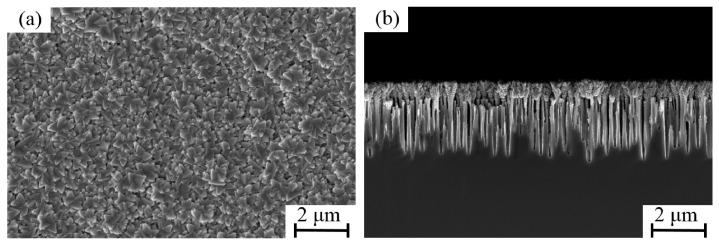
Surface (**a**) and cross-sectional (**b**) SEM images of getter films after annealing at 450 °C.

Figure 3 illustrates the growth process of the Ti film on the black silicon substrate. At the initial stage, the film exhibits a uniformly distributed spherical morphology. The morphology of the subject is relatively smooth, characterized by clearly defined interstitial spaces between adjacent spheres. In addition, cross-sectional SEM images reveal that the spherical structures predominantly nucleate and grow at the tips of the nanoneedles (Figure 3a,b). As the process of deposition continues, the radius of the spheres undergoes a gradual increase. As the deposition time increases, the surfaces of the spheres become rougher, and the spacing between neighboring spheres decreases (Figure 3c,d). As the deposition process reaches its conclusion, the spherical structures undergo a transformation, assuming an inverted cone-like morphology. Concurrently, the interstitial spaces between the spheres become obscured (Figure 3e,f). The deposition of Ti getter films was undertaken utilizing the same deposition parameters on conventional planar silicon substrates as well. As shown in Figure 4, the surface morphology of the film is characterized by the uniform distribution of block-like stacked structures. The cross-sectional SEM images suggest that the film is relatively dense, with no discernible pores. To further evaluate the thermal stability of the black silicon nanostructure, a more stringent validation strategy was intentionally adopted. Specifically, samples with longer deposition time (60 min) were examined, in which the Ti film evolves into a more continuous and less porous morphology. Such structures are expected to induce higher thermal stress during annealing. As shown in Figure 5, no evident structural collapse, tip coalescence, or morphology degradation was observed after annealing at 450 °C. This result indicates that the black silicon scaffold maintains good structural integrity even under more demanding conditions, suggesting that the porous structures presented in this work are sufficiently stable during activation. In addition, the mechanical stability of the porous structure is supported by the deposition process and structural configuration. The Ti layer was deposited by direct sputtering onto the silicon substrate, thereby ensuring excellent adhesion. The three-dimensional nanostructure of the layer offers additional mechanical interlocking, which is beneficial for maintaining structural integrity in practical MEMS applications.

**Figure 6:**
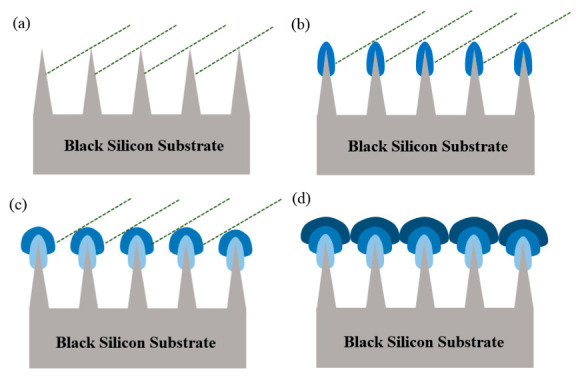
Principles of thin film deposition on black silicon substrates: (**a**) mutual shadowing effect in the initial stage; (**b**) preferential growth at tips; (**c**) self-amplifying shadowing effect; (**d**) formation of inverted cone-like structures in the final stage.

Figure 6 illustrates the formation mechanism of the inverted cone-like structures. Owing to the inclined geometry between the sputtering target and the substrate in the sputtering system, a mutual shadowing effect occurs between adjacent nanoneedles (Figure 6a). Consequently, a negligible proportion of the sputtered atoms are able to reach the bottom regions of the black silicon structures, with film growth predominantly occurring at the tops of the nanoneedles, forming spherical structures (Figure 6b). These spherical features further enhance the shadowing effect, thereby enabling the film growth to continue primarily at the tops of the black silicon structures (Figure 6c). In the course of subsequent deposition, the film growth rate in the regions beneath the nanoneedles progressively decreases, while the regions above the nanoneedles exhibit a relatively stable growth rate. This differential growth ultimately leads to the formation of an inverted cone-like porous structure (Figure 6d). In this study, the shadowing effect is discussed in a qualitative manner based on the observed morphological evolution at different deposition stages. Despite the absence of a systematic modelling of quantitative parameters such as incident angle, deposition rate, and structure aspect ratio, the sequential SEM results (Figure 3) provide unequivocal experimental evidence for the transition from isolated nucleation to tip-enhanced growth and finally to inverted cone-like porous structures, which is consistent with a shadowing-dominated growth mechanism.

**Figure 7:**
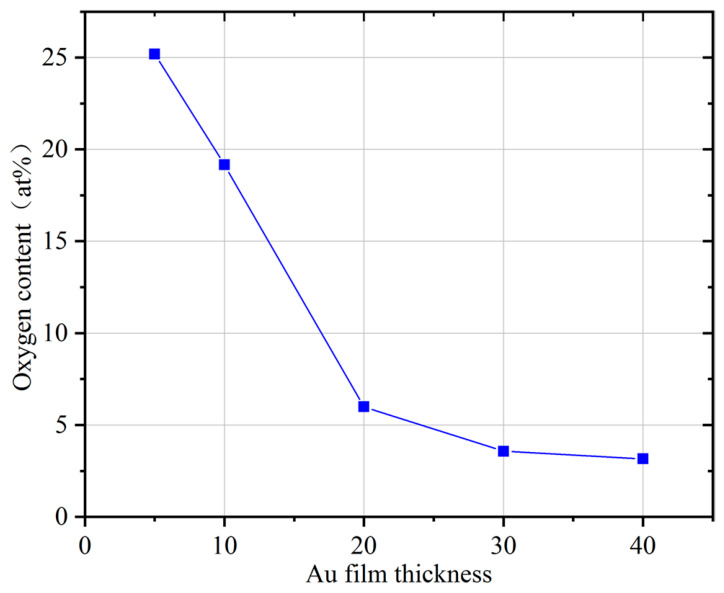
Relationship between film surface oxygen content and Au film thickness.

Ti is highly susceptible to oxidation in air. When integrated into the cavity of MEMS wafer-level packages, such oxidation can severely degrade the sorption capability of the getter film. To prevent this oxidation, it is therefore necessary to deposit a gold film in situ as a protective layer. Ideally, the Au film should be as thin as possible while still providing sufficient coverage of the surface of the inverted cone-like porous structures. In this work, XPS was employed to assess the surface oxygen content, with the objective of optimising the thickness of the Au protective layer. The results are shown in Figure 7. When the Au film thickness was 5 nm, a considerable amount of oxygen was detected on the film surface. This indicated that the Au layer could not form a continuous coating on the rough and porous Ti surface. As the thickness of the Au film increased, the oxygen content decreased significantly. When the Au thickness is 10 nm, the surface still shows a relatively high oxygen content (~19%), indicating insufficient protection of the underlying Ti layer. It is evident that augmenting the thickness to 20 nm results in a substantial diminution of the oxygen content to approximately 6%, thereby indicating that the majority of the Ti layer is effectively safeguarded. However, further increases in the Au thickness, up to 30 nm, result in only a slight additional reduction in oxygen content (~3.6%), indicating a diminishing improvement. On the other hand, the presence of a more substantial Au layer could impede the diffusion of Ti during the activation process. This could necessitate elevated activation temperatures or protracted activation times, a circumstance that is unfavorable for MEMS applications characterized by constrained thermal budgets. Consequently, the selection of 20 nm is regarded as a reasonable compromise rather than a strict optimum. The same measurements were also performed on Ti films deposited on conventional flat silicon substrates. The results indicate that the optimal thickness of the Au protective layer in this instance is 5 nm, thereby further substantiating the porous nature of the getter film.

**Figure 8:**
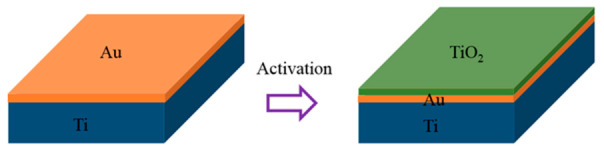
Interdiffusion process of Au and Ti during activation.

**Figure 9:**
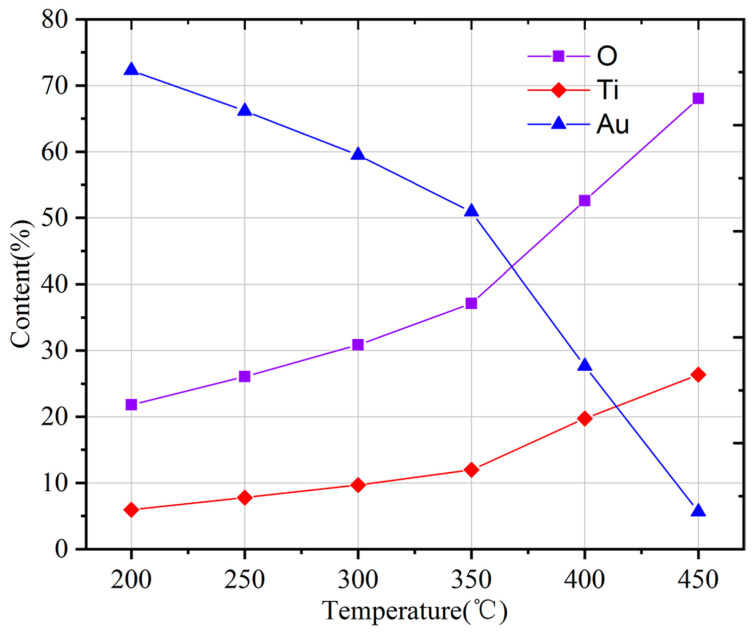
Variation in O, Ti, and Au content with temperature at the film surface.

**Figure 10:**
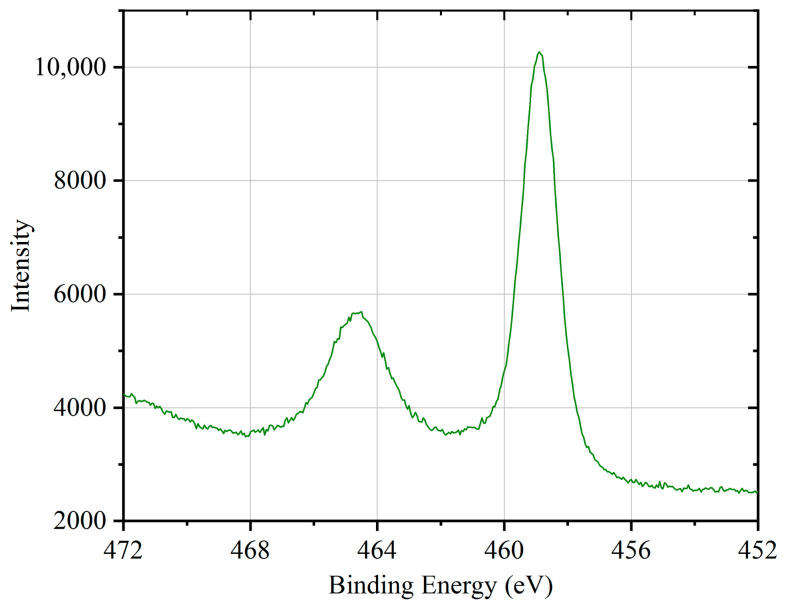
XPS Ti 2p peak of the getter film after activation at 450 °C.

The activation of the Au/Ti bilayer getter film occurs through the outward diffusion of Ti atoms through the Au layer (Figure 8). Previous studies have reported that Ti atoms can diffuse through Au layers at temperatures above 200–400 °C, leading to surface segregation and oxidation [13,28]. In view of the limitations of the thermal budget and the necessity to regulate thermomechanical stress in MEMS devices, it is recommended that the practical activation temperature should be maintained below 450 °C [29]. To evaluate the sorption capability of the Au/Ti bilayer films activated at different temperatures, the annealing temperature was varied from 200 °C to 450 °C with a duration of 1 h. Following the process of annealing, the samples were retrieved from the vacuum system and exposed to an air environment in a cleanroom setting for a period of 24 h prior to the execution of XPS measurements. The exposure was conducted at ambient temperature (approximately 25 °C) under natural laboratory humidity (approximately 40%), without the application of additional environmental control measures. It is important to note that, due to unavoidable air exposure during sample transfer, the present study focuses on the relative changes in surface chemical states under identical exposure conditions. This approach has been extensively employed in the field of thin-film getter studies to assess activation behavior and sorption performance [30]. Figure 9 shows the variation in the total amounts of O, Ti, and Au detected in the exposed films as a function of annealing temperature. It is evident that there is a substantial increase in the oxygen content of the films with an increase in the annealing temperature. Moreover, this enhancement does not demonstrate a linear dependence on temperature, suggesting that elevated annealing temperatures facilitate the accumulation of Ti atoms at the free surface. This interpretation is further substantiated by the concomitant decrease in the Au concentration detected on the film surface. It is noteworthy that Au can still be detected even after annealing at 450 °C. Considering the probing depth of XPS (approximately 2–5 nm), these Au signals are attributed to residual Au atoms remaining after diffusion along grain boundaries. Figure 10 shows the Ti 2p spectrum at an annealing temperature of 450 °C. This spectrum exhibits a Ti 2p_3/2_ peak located at 458.8 eV and a Ti 2p_1/2_ peak at 464.5 eV. These peaks are consistent with the high-valence oxide TiO_2_. This result indicates that the Ti present on the film surface has undergone extensive oxidation. Therefore, it can be deduced that the getter film has been successfully activated and is suitable for application in vacuum packaging. In addition to the Au/Ti system investigated in this study, the proposed three-dimensional black silicon scaffold can be expanded to encompass other thin-film getter materials. This provides a comprehensive approach for enhancing the effective surface area in MEMS-compatible getter designs. In future work, system-level validation will be carried out through wafer-level packaging integration and testing platforms to assess the long-term stability and gas sorption performance of the proposed structure in practical MEMS applications.

## 4. Conclusions

A porous Au/Ti bilayer thin-film getter based on a black silicon substrate has been developed for the purpose of MEMS vacuum packaging. The Au layer, with a thickness of 20 nm, serves as a protective layer to prevent oxidation of the getter film while maintaining effective activation behavior. The fabrication of the black silicon nanostructure is achieved by ICP etching, a process which provides a high-surface-area three-dimensional scaffold without the necessity for additional lithography steps. It has been demonstrated that the shadowing effect between adjacent nanoneedles leads to the formation of an inverted cone-like porous morphology in the Ti film, thereby resulting in a substantial enhancement in the effective surface area of the getter film. XPS analysis confirms that, following annealing at 450 °C, Ti atoms diffuse to the surface and form TiO_2_ upon air exposure, thereby indicating successful activation of the getter. This work demonstrates a simple and MEMS-compatible strategy for constructing porous thin-film getters based on a three-dimensional black silicon scaffold, providing an effective approach to enhance surface area through shadowing-induced growth.

## Figures and Tables

**Table 1:** Etching parameters of black silicon.

Power (W)	Pressure (mTorr)	O_2_ (sccm)	SF_6_ (sccm)	Etching Time (min)
1500	30	60	42	5

**Table 2:** Deposition parameters of getter films.

Material	Sputtering Power (W)	Sputtering Pressure (mTorr)	Ar Flow Rate (sccm)	Deposition Rate (nm/min)
Ti	250	5	30	13.5
Au	250	5	30	25

## Data Availability

The data presented in this study are available on request from the corresponding author.
